# Effect of organic-inorganic combined application on rice yield and nitrogen fate in soda saline alkali soil

**DOI:** 10.3389/fpls.2026.1806937

**Published:** 2026-10-29

**Authors:** Yan Bao, Yang Li, Yue Zhao, Zhaofeng Song, Meijun Li, Yu Han, Shuyao Guo, Xiao-yan Zhang, Xiaoshu Chen

**Affiliations:** 1 Jilin Agriculutral Science and Technology College, Jilin, China; 2 Jilin Academy of Agricultural Sciences/China Agricultural Science and Technology Northeast Innovation Center, Changchun, China; 3 Changbai Mountain Nature Conservation and Management Center, Yanbian, China

**Keywords:** nitrogen loss, nitrogen utilization efficiency, organic-inorganic combination application, rice, soda saline alkali soil

## Abstract

In soda saline-alkali soil, long-term excessive application of inorganic nitrogen fertilizer leads to low nitrogen use efficiency, severe nitrogen loss, and aggravated soil degradation, which seriously restricts rice production and regional food security. Organic fertilizer, with its advantages of improving soil structure and providing slow-release nutrients, has potential in optimizing rice nitrogen utilization and alleviating environmental risks. This study took rice in soda saline-alkali soil as the research object, a set of 6 treatments including no nitrogen fertilization (N0), single application of chemical fertilizers (NPK), and 4 organic nitrogen substitution ratios (25%M, 50%M, 75%M, M (100%M)) with pig manure as organic fertilizer source, and measured indicators such as rice yield, dry matter accumulation, nitrogen accumulation, nitrogen use efficiency, soil inorganic nitrogen residue, and nitrogen loss (ammonia volatilization, N_2_O emission) during 2024-2025. Results showed that compared with CK (2978 kg ha^-1^), all fertilization treatments significantly increased rice yield by 77.1% - 117.9%, and organic substitution treatments (except M) increased yield by 4.3%-8.9% compared with NPK (5485 kg ha^-1^), with 50%M treatment having the highest yield (6389 kg ha^-1^). Organic substitution treatments (except M) increased rice aboveground nitrogen accumulation by 19.8%-22.9% and apparent nitrogen utilization efficiency by 10.3-12.4 percentage points compared with NPK, and 50%M treatment had the highest nitrogen accumulation (150.6 kg ha^-1^) and apparent nitrogen utilization efficiency (34.0%). Compared with NPK, organic substitution treatments reduced soil 0-100cm inorganic nitrogen residue by 11.3%-38.0% and cumulative ammonia volatilization by 12.3%-19.98%, with 50%M treatment having the lowest soil inorganic nitrogen residue (49.8-54.6 kg ha^-1^) and nitrogen loss rate (38.51%). Excessive organic substitution (M treatment) led to lower yield and nitrogen use efficiency than 50%M, with yield even not significantly different from NPK. This confirms that 50% organic nitrogen substitution with pig manure is significant for improving rice yield, nitrogen use efficiency, and reducing nitrogen loss in soda saline-alkali soil.

## 1 Introduction

As an important global food crop, the stable production of rice is critical to ensuring global food security (Ladha et al., 2021; Wei et al., 2022; Yamini et al., 2025). China’s Northeast Plain, endowed with vast arable land resources and favorable climatic conditions, has developed into a major grain-producing region in the country. Both the planting area and yield of rice have been on a steady increase year by year, playing an indispensable role in safeguarding the national food security strategy (Hou et al., 2025). According to statistics, the rice planting area in the three northeastern provinces has exceeded 7 million hectares, with an annual output of over 40 million tons, accounting for approximately 1/5 of China’s total rice production (Dong et al., 2023; Xiao et al., 2024). However, the total area of saline-alkali land in Northeast China reaches as high as 3.6 million hectares, mainly distributed in the Songnen Plain, Liaohe Plain and other regions. The soil pH value generally ranges from 8.5 to 9.5, with a salt content of 0.3% - 1.0% and an alkalinity exceeding 15%. Saline-alkali soils are characterized by high pH, high salinity, and low organic matter content (Liang et al., 2008). The excessive presence of sodium ions damages the soil particle structure, resulting in soil compaction and poor permeability (Camelo and Duval, 2025). A high pH environment significantly reduces the availability of nutrients such as phosphorus, iron, and zinc, which not only impairs rice root growth and nutrient absorption but also leads to the deterioration of soil structure, seriously hindering the normal physiological metabolism of rice (Singh and Singh, 2005; Kobayashi et al., 2010; Hui et al., 2017). Previous studies have demonstrated that in severely saline-alkali lands, rice yield reduction caused by salt stress can reach 30% - 50%, posing a significant threat to regional food production security (Tack et al., 2015; Dam et al., 2019; Li et al., 2023).

To mitigate the adverse effects of saline-alkali land on rice production, chemical nitrogen fertilizers have long been widely used in rice cultivation in the saline-alkali regions of Northeast China (Mehdi et al., 2007). The annual application of nitrogen fertilizers in Chinese farmland has exceeded 25 million tons, with a unit area application rate of 225 kg/ha, which is far higher than the global average level (80 kg/ha). However, since farmers still mainly rely on inorganic nitrogen fertilizers, the seasonal nitrogen use efficiency is generally low, mostly below 40% (Chai et al., 2019). The application rate of inorganic nitrogen fertilizers in saline-alkali paddy fields is 20% - 30% higher than that in conventional soils, while the nitrogen accumulation in rice grains only increases by 8% - 12% (Li et al., 2025). Although excessive application of inorganic nitrogen fertilizers can increase rice yield in the short term, it has caused a series of negative impacts. On the one hand, a large amount of nitrogen unabsorbed by rice remains in the soil, altering the balance of soil nitrogen forms and leading to increased soil acidification and salinization (Yang et al., 2020). Long-term positioning experiment data show that after 10 consecutive years of excessive nitrogen fertilizer application, the average pH value of saline-alkali soils in Northeast China decreased by 0.8-1.2 units, and the accumulation of nitrate nitrogen increased by 2–3 times, exacerbating the risk of secondary soil salinization (Han et al., 2015; Zhang et al., 2022). On the other hand, nitrogen is largely lost through pathways such as ammonia volatilization and leaching, which not only results in resource waste but also causes environmental problems such as water eutrophication (Peng et al., 2015; Carlos et al., 2023). In the unique soil environment of saline-alkali land in Northeast China, high pH conditions significantly accelerate the ammonia volatilization process of ammonium nitrogen (Dendooven et al., 2010). Studies have shown that when the soil pH is higher than 8.5, the ammonia volatilization rate is 2–3 times higher than that in neutral soil; poor soil aeration is prone to cause denitrification loss of nitrate nitrogen, especially in the flooded environment from the tillering stage to the booting stage of rice. Denitrification loss accounts for 15% - 25% of the total nitrogen application, making the problem of nitrogen loss more prominent (Ma et al., 2024). As a traditional and efficient fertilizer, organic fertilizer contains abundant organic matter, humus, and various nutrients, and has unique advantages in improving soil structure, enhancing soil fertility, and promoting crop growth (Yu and Ren, 2015; Tian et al., 2022). Taking cow manure and straw compost as examples, their organic matter content is usually between 30% - 50%, total nitrogen content is 1.5% - 3.0%, and they contain a large number of organic nitrogen components such as amino acids and peptides (Islam and Karim, 2011). Research has indicated that after organic fertilizer is applied to the soil, the organic matter it contains can be decomposed and converted into humus by microorganisms, improving the soil particle structure, enhancing the soil’s water and fertilizer retention capacity, and thereby promoting the absorption of nitrogen by rice roots (Albiach et al., 2001). Meanwhile, nitrogen in organic fertilizers mostly exists in organic form and needs to be gradually released through microbial mineralization. This slow-release characteristic can better match nitrogen supply with the growth needs of rice, theoretically improving nitrogen use efficiency and reducing nitrogen loss (Ve et al., 2004).

However, current research on organic fertilizer substitution for inorganic fertilizers in rice production in the saline-alkali soils of Northeast China still has significant limitations. Although preliminary studies have explored the effects of organic fertilizers on soil improvement and crop growth in saline-alkali land, most of them have focused on single substitution ratios or short-term experiments, lacking systematic research on the patterns of rice nitrogen absorption, residue, and loss under different substitution ratios. Therefore, based on the actual demand for rice production in the saline-alkali land of Northeast China, this study systematically investigates the effects of organic fertilizer on rice nitrogen absorption, greenhouse gas emissions, soil nitrogen residue, and other aspects by setting different proportions (0%, 20%, 40%, 60%, 80%) of organic fertilizer substitution for inorganic fertilizer treatments. The objectives are to clarify the dynamic changes of rice nitrogen absorption under different substitution ratios, reveal the characteristics of soil nitrogen residues and their potential environmental risks, quantify the flux differences of major nitrogen loss pathways such as ammonia volatilization, and elucidate the underlying mechanisms driving these changes. The research results will provide a theoretical basis for scientific and rational fertilization in rice production in the saline-alkali land of Northeast China, and contribute to the efficient utilization of saline-alkali land resources and the green and sustainable development of agriculture.

### 1.1 Basic information of the experimental area

The experimental site is located in Wuyi Village, Tongyu County, Jilin Province, with a soil type ofsoda saline-alkali soil. Collect 0-20cm topsoil before the experiment and determine its basic physical and chemical properties: pH value 8.9 (soil water ratio 1:2.5, m/v); Electrical conductivity (EC) 0.82 mS cm^-1^; Total salt content 0.45 g kg^-1^; Na^+^ content 38.6 mg kg^-1^; HCO_3_^-^ content 125.3 mg kg^-1^; Soil organic matter content is 6.2g kg^-1^; Alkaline hydrolyzed nitrogen content 42.8mg kg^-1^; The content of available phosphorus (Olsen-P) is 11.5mg kg^-1^; The content of available potassium (NH_4_OAc-K) is 108.3 mg kg^-1^; The effective sulfur application rate is 7.8mg kg^-1^, the bulk density is 1.42g cm^-3^, and the maximum field water holding capacity is 25.3%; Electrical conductivity (EC): 0.82 mS cm^-1^; total salt content: 0.45 g kg^-1^.

### 1.2 Experimental design

The experiment consisted of 6 treatments with three replications: no nitrogen fertilization (N0), single application of chemical fertilizers (NPK), and four treatments with pig manure replacing 25%, 50%, 75%, and 100% of chemical nitrogen, referred to as 25%M, 50%M, 75%M, and M (100%M), respectively. Application rates of N, P_2_O_5_, K_2_O, and pig manure for each treatment are summarized in **Table 1**.

**Table 1:** Application rates of N, P_2_O_5_, K_2_O, and pig manure under different treatments.

Treatment	N (kg ha^-1^)	P_2_O_5_ (kg ha^-1^)	K_2_O (kg ha^-1^)	Pig manure (kg ha^-1^)
N0	0	75	150	0
NPK	150	75	150	0
25%M	150	75	150	3125
50%M	150	75	150	6250
75%M	150	75	150	9375
M	150	75	150	12500

Fertilizer sources: Urea (N 46%), calcium magnesium phosphate (P_2_O_5_ 12%), and potassium chloride (K_2_O 60%) were conventional agricultural fertilizers purchased from a local market. Pig manure was obtained from a standardized breeding farm in Jilin Province, subjected to aerobic composting for 45 days, and processed into commercial organic fertilizer meeting the NY525–2011 standard. Key properties of pig manure were as follows: pH 8.09, organic carbon 33.12%, total N 1.20%, total P (P_2_O_5_) 1.36%, total K (K_2_O) 1.46% (dry basis). Heavy metal contents were Cu 146.8 mg kg^-1^, Zn 215.3 mg kg^-1^, Cd 0.28 mg kg^-1^, Pb 8.6 mg kg^-1^, all below the maximum limits specified in NY525-2021.

Rice cultivar is Changbai 9, a salt-alkali tolerant variety widely planted by local farmers in soda saline-alkali soil of Northeast China, was used in this study.Except for N0, all treatments followed the equivalent nutrient principle with N, P_2_O_5_, and K_2_O rates of 150, 75, and 150 kg ha^-1^, respectively. The application rate of pig manure was calculated based on total nitrogen content, and P and K nutrients supplied by pig manure were deducted from chemical fertilizers. Pig manure, phosphorus, and potassium fertilizers were applied as basal fertilizer in one time. Chemical nitrogen fertilizer was applied in three splits: 40% as basal fertilizer, 30% at tillering stage, and 30% at booting stage. Each plot was 40 m² (5 m × 8 m), and plots were separated by 50 cm buffer zones with plastic film to avoid water and fertilizer cross-flow. The experiment was conducted in 2024 and 2025, with a single rice crop per year. Rice planting density and field management were consistent with local practices.

### 1.3 Measurements and methods

#### 1.3.1 Rice yield

According to the method described by Parida et al. (2025), during the rice maturation stage, 50 randomly selected panicles were surveyed in each plot to calculate the effective number of panicles. The actual yield was determined by harvesting a 5 m^2^ area from the central zone of each plot, and the actual yield was calculated at a standard grain moisture content of 13.5%.

#### 1.3.2 Plant nutrient accumulation measurement

During the mature stage of plant sample collection, three evenly growing rice plants were selected from each plot and cut from their roots. They were then placed in mesh bags and brought back to the laboratory for air drying treatment. All plant samples were dried and ground at 70 °C for nitrogen content determination. Following the methodology proposed by Sun et al. (2012), using H_2_SO_4_-H_2_O_2_ combined digestion, the total nitrogen of the plant was measured using a Kjeldahl nitrogen analyzer. The nitrogen accumulation is calculated by multiplying the nitrogen content of each part of the plant with its dry matter mass.

#### 1.3.3 Determination of inorganic nitrogen and moisture content in soil

The method of collecting soil samples was as follows: a soil drill was used to excavate soil samples from 0 to 100 cm of the soil profile in layers during the mature stage. The soil profile was divided into five layers, namely 0–20 cm, 20–40 cm, 40–60 cm, 60–80 cm, and 80–100 cm. Three sampling points were set for each layer, and the soil samples from the same layer were mixed thoroughly. All the collected fresh soil samples were stored in the same refrigerated container, and then passed through a 4–5 mm sieve for homogenization before subsequent analysis. A 12.00 g aliquot of the mixed soil sample was weighed and mixed with 100 mL of 0.01 mol/L CaCl_2_ leaching solution. The mixture was shaken at 180 rpm for 30 minutes, followed by filtration and freezing of the filtrate. Meanwhile, a 15.00 g soil sample was taken to determine the soil moisture content. The filtrate was measured using a TRAAS-2000 continuous flow analyzer (Manufacturer: Beijing Taioo Technology Co., Ltd., Beijing, China).

#### 1.3.4 N_2_O、NH_3_ monitoring and environmental indicator measurement method

##### 1.3.4.1 N_2_O emission monitoring method

Referring to the method of Cosme et al. (2015), N_2_O was detected using static box gas chromatography. The sampling box includes a stainless steel base and a top box. The bottom is a tetrahedral shape of 50 cm x 50 cm x 30 cm, with a circular hole on the lower side for normal exchange of energy and matter between the inside and outside. The top box is a square cube with a length of 50 cm, a width of 50 cm and a height of 50 cm, which is made of stainless steel. The outside is insulated with foam board. There are two fans inside for mixing air, and there is also a temperature detector and a sampling pipe. In order to reduce soil disturbance on N_2_O emissions, the base was seeded and fertilized on the pedestal one week after sowing.

The sampling frequency is mainly concentrated after fertilization, and gradually decreases thereafter. If there are fertilization, precipitation and other situations, the sampling frequency increases. After fertilization, once a day for 10 consecutive days until N_2_O emissions stabilize; After rainfall (rainfall>10mm), for 7 consecutive days, once a day, until N2O emissions reach normal levels. Each sample is taken from 9:00 am to 11:00 am on the same day. At 0, 10, 20, and 30 minutes after opening the box, 50 ml of gas is extracted using a 60 ml syringe equipped with a three-way valve. The sample is then stored in a syringe and analyzed on the same day.

The concentration of N_2_O in the sample was determined using a gas chromatograph with an ECD detector (Agilent 7890 A, Shanghai, China). The carrier gas is 99.999% high-purity nitrogen gas, with a flow rate of 21 ml min^-1^. The temperature of the detector and column box are 300 °C and 60 °C, respectively.

The N_2_O emission flux is calculated using the following formula:


F=P/1013×28/22.4×273/(273+T)×H×dc/dt×60

Among them, F is the N_2_O emission flux (μ g N_2_O-N m^-2^ h^-1^), P is the atmospheric pressure during gas collection (h Pa), T is the average temperature inside the box during gas collection (°C), H is the height of the sampling box (m), and dc/dt is the rate of N_2_O concentration change during the sampling time (μl L^-1^ min^-1^, calculated by linear regression equation). The cumulative emissions of N_2_O are calculated using linear interpolation method.

Measurement of environmental and soil parameters:

When installing at the bottom of the sampling box, place two thermometers on the surface of the sampling box and at a depth of 10 cm. Use an electronic thermometer to measure the temperature inside the box, on the surface, and at a depth of 10 cm from the soil during sampling, and take the average of the two readings as the average temperature during the sampling process. In the subsequent analysis, the soil temperature of each treatment was based on the average temperature of the surface layer and 10 cm depth.

During the sampling process, the soil drilling method is used to take soil samples from 0–20 cm outside the frame to measure the soil moisture content and inorganic nitrogen content. Meanwhile, the collection frequency of nitrous oxide is also the same. After sampling, soil moisture and inorganic nitrogen should be tested immediately. The moisture content is determined by drying method, while inorganic nitrogen is measured by CaCl_2_ leaching continuous flow analyzer.

##### 1.3.4.2 Soil ammonia volatilization

Using the semi aeration method, samples were collected and tested on the 1st, 2nd, 3rd, 4th, 5th, 6th, 7th, 9th, 11th, 13th, 16th, 19th, 22nd, 29th, 36th, 43rd, 58th, 73rd, and 88th days after fertilization until no significant NH_3_ volatilization was detected. 0.1 mol L^-1^ KCl solution was used for oscillatory extraction, and the ammonium nitrogen content was determined by a flow analyzer.

##### 1.3.4.3 Soil CH_4_ emissions

Refer to the method proposed by Martin et al (Krüger and Frenzel, 2010). The soil CH_4_ emission flux was measured using a static closed box gas chromatography method, which is widely used in *in-situ* monitoring of greenhouse gases in paddy ecosystems. The sampling system is consistent with the device used for N_2_O determination. The gas sampling is conducted from 9:00 a.m. to 11:00 a.m. on a sunny day, and it will be postponed in case of a rainstorm. The sampling frequency should be consistent with the N_2_O measurement: after fertilization and rainfall, the sampling should be intensified, specifically once a day for 10 consecutive days after fertilization, until the CH_4_ emission flux stabilizes; After the rainfall exceeds 10mm, once a day for 7 consecutive days until the emissions return to normal levels. During each sampling, place the top box on the pre installed base, and after the box is sealed for 0, 10, 20, and 30 minutes, use a 60 mL syringe with a three-way valve to extract 50 mL of gas sample. All samples are stored in syringes and transported back to the laboratory for analysis on the same day.

The concentration of CH_4_ in the sample was determined using a gas chromatograph (Agilent 7890A, Shanghai, China) equipped with a hydrogen flame ionization detector (FID). The carrier gas is 99.999% high-purity nitrogen with a flow rate of 21 mL · min^-1^; the detector temperature and column box temperature are set to 200 °C and 55 °C, respectively. The CH_4_ emission flux is calculated using the following formula based on the linear variation of gas concentration during the sampling period (correlation coefficient R >0.90):


$$F\;=\;\rho \;\times \;h\;\times \;\left(dc/dt\right)\;\times \;\left(273/\left(273\;+\;T\right)\right)$$

In the formula: F is the CH_4_ emission flux (mg · m^-3^ h^-1^); ρ is the CH_4_ density under standard conditions (kg m^-3^); H is the net height of the sampling box (i.e. the distance from the top of the box to the water surface, m); dc/dt is the rate of change of CH_4_ concentration in the box over time; 273 is the constant of the gas state equation; T is the average temperature inside the box during the sampling period (°C).

Gas sampling was synchronized, and an *in-situ* pH/mV meter (pH 3110, WTW, Wilhelm, Germany) was used to measure the soil redox potential (Eh) and soil temperature at a depth of 10 cm, recording environmental factors that affect CH_4_ emissions. Each community’s various indicators are measured three times to ensure data reliability.

#### 1.3.5 Formula calculator

Calculate nitrogen utilization efficiency, nitrogen residue rate, nitrogen loss rate, nitrogen partial productivity, and nitrogen agronomic efficiency using the method proposed by Hadush et al (HadushN et al., 2020). Apparent residual rate of nitrogen fertilizer (%)=(residual inorganic nitrogen in soil in nitrogen application area - residual inorganic nitrogen in soil without nitrogen application area)/nitrogen application rate x 100;

The apparent utilization rate of nitrogen fertilizer (%)=(nitrogen uptake by crops in the nitrogen application area - nitrogen uptake by crops in the non nitrogen application area)/nitrogen application amount x 100;

The apparent loss rate of nitrogen fertilizer (%) is 100% - the apparent utilization rate of nitrogen fertilizer - the apparent residual rate of nitrogen fertilizer.

$Nitrogen\;Fertilizer\;Productivity\left(kg/kg\right)=Y_{N}/N_{a}$ $Agronomic\;Efficiency=\left(Y_{N}-Y_{0}\right)/N_{a}$In the formula, YN is the grain yield of crops treated with nitrogen, and Y0 is the grain yield of crops without nitrogen blank control; Na is the amount of nitrogen applied.

### 1.4 Data analysis

Microsoft Office Excel 2017 (Microsoft Windows, Redmond, USA) was used to process data and charts, and analysis of variance (ANOVA) was performed using SPSS software (IBM Statistics 21.0, New York, USA). The LSD method was employed to compare the differences between two treatments, with a significance level of *P* = 0.05.

## 2 Results and analysis

### 2.1 Effect of different organic substitution ratios on grain yield

From **Figure 1**, it can be seen that over the course of two years, compared to N0 processing, each treatment significantly increased rice yield compared to N0 (2024:3268kg ha^-1^; 2025:2688kg ha^-1^), with an average increase of 77.1% -117.9%. And the yield of each organic substitution treatment was significantly increased compared to the NPK treatment (2024:5354kg ha^-1^; 2025:5615kg ha^-1^), with an average increase of 4.3% -8.9%. And there is no difference in yield between M treatment and NPK treatment. Among all organic substitution treatments, the rice yield with 50%M was the highest (2024:6367kg ha^-1^; 2025: 6410kg ha^-1^), with an average increase of 13.0% -13.8% compared to other organic substitution treatments.

**Figure 1:**
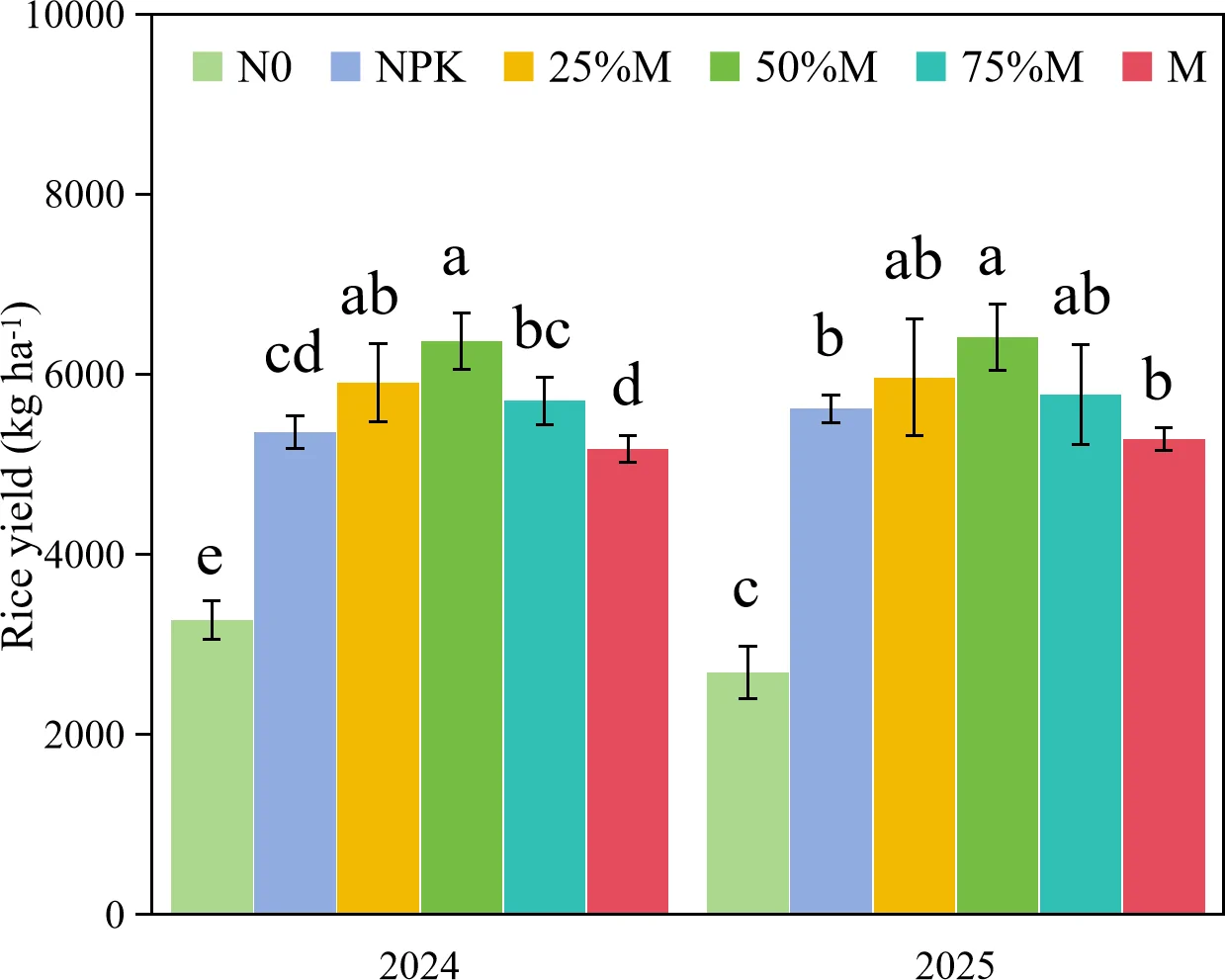
Effect of different organic substitution ratios on rice yield. Values are the means with standard deviations shown by vertical bars (n = 3). N0 represents no nitrogen fertilizer treatment; M represents organic fertilizer replacing 25% N; 50% M represents organic fertilizer replacing 50% N; 75% M represents organic fertilizer replacing 75% N; M treatment represents complete application of organic fertilizer; The lowercase letters “a, b, c, d” indicate significant differences (*P*< 0.05) between different treatments.

### 2.2 Effect of different organic substitution ratios on accumulation of dry matter

From **Figure 2**, it can be seen that different nitrogen fertilizer applications can significantly increase the above ground dry matter accumulation of rice at maturity stage over two years, compared to N0 processing, the average improvement of each organic substitution treatment was 18.8% -46.0%. In 2025, all treatments except for N0 treatment increased by an average of 2.8% compared to 2024. Other organic substitution treatments, except for treatment M, significantly increased the aboveground dry matter accumulation of rice compared to NPK treatment, with an average increase of 9.5% -10.7% over two years. In the organic substitution treatment, the rice with 50% M treatment had the highest dry matter accumulation in the aboveground part (2024: 12812kg ha^-1^; 2025: 13452 kg ha^-1^), which increased by 11.3% -13.2% compared to other organic substitution treatments.

**Figure 2:**
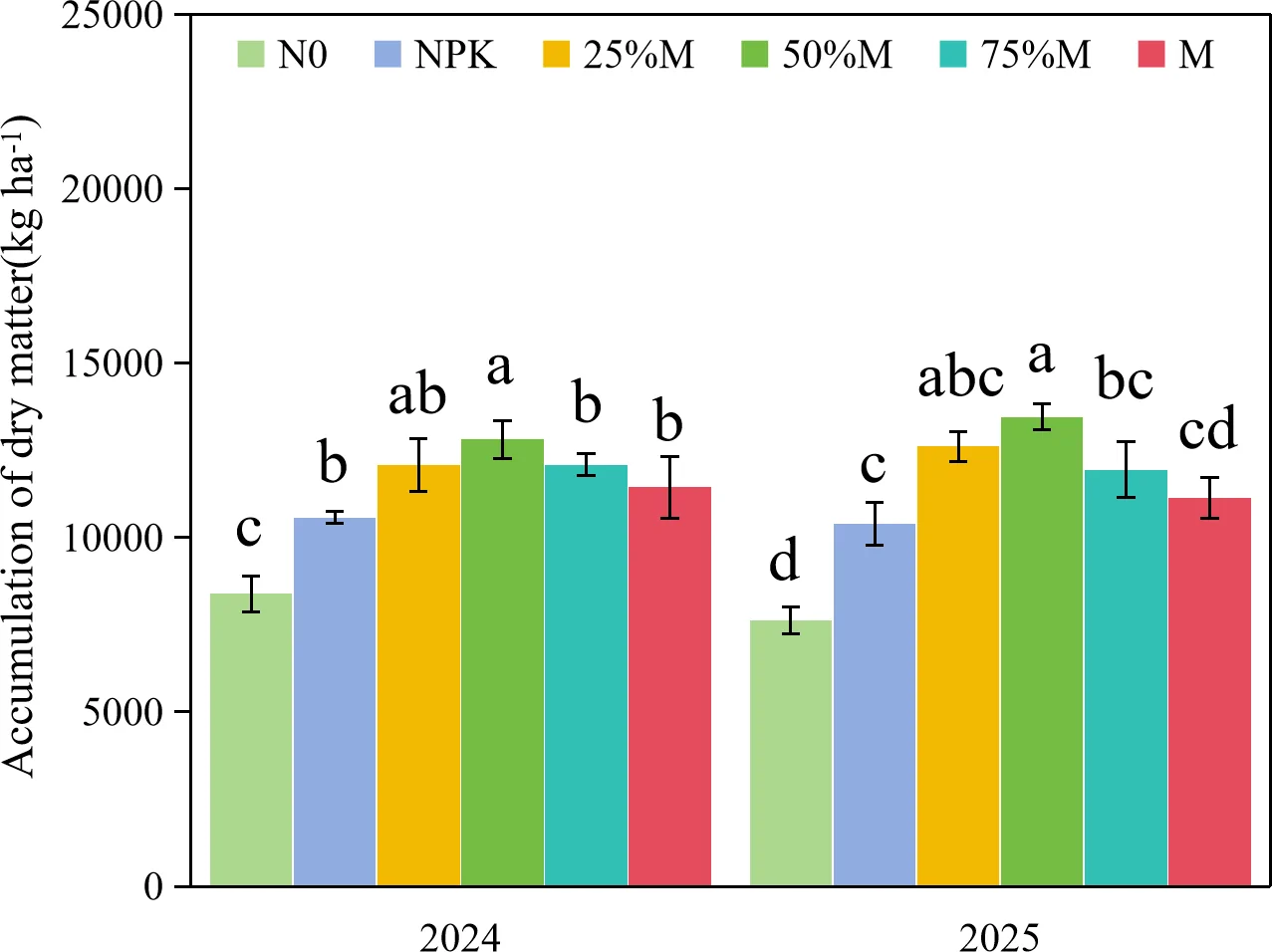
Effect of different organic replacement ratios on aboveground biomass of rice in soda saline alkali soil. Values are the means with standard deviations shown by vertical bars (n = 3). N0 represents no nitrogen fertilizer treatment; M represents organic fertilizer replacing 25% N; 50% M represents organic fertilizer replacing 50% N; 75% M represents organic fertilizer replacing 75% N; M treatment represents complete application of organic fertilizer; The lowercase letters “a, b, c, d” indicate significant differences (*P*< 0.05) between different treatments.

### 2.3 Effcet of different organic substitution ratios on nitrogen accumulation

From **Figure 3**, it can be seen that different nitrogen fertilizer applications can significantly increase the nitrogen accumulation in the aboveground part of rice during the mature stage over two years, with an average increase of 74.3% -141.7%. In 2025, all treatments except for N0 treatment showed a significant increase of 8.2% compared to the average of 2024. Other different organic substitution treatments, except for treatment M, significantly increased the nitrogen accumulation in the aboveground part of rice compared to NPK treatment, with an average increase of 19.8% -22.9% over two years. In the organic substitution treatment, the rice with 50% M treatment had the highest aboveground biomass (2024: 147.1 kg ha^-1^; 2025: 154.0 kg ha^-1^), which increased by 12.6% -19.3% compared to other organic substitution treatments.

**Figure 3:**
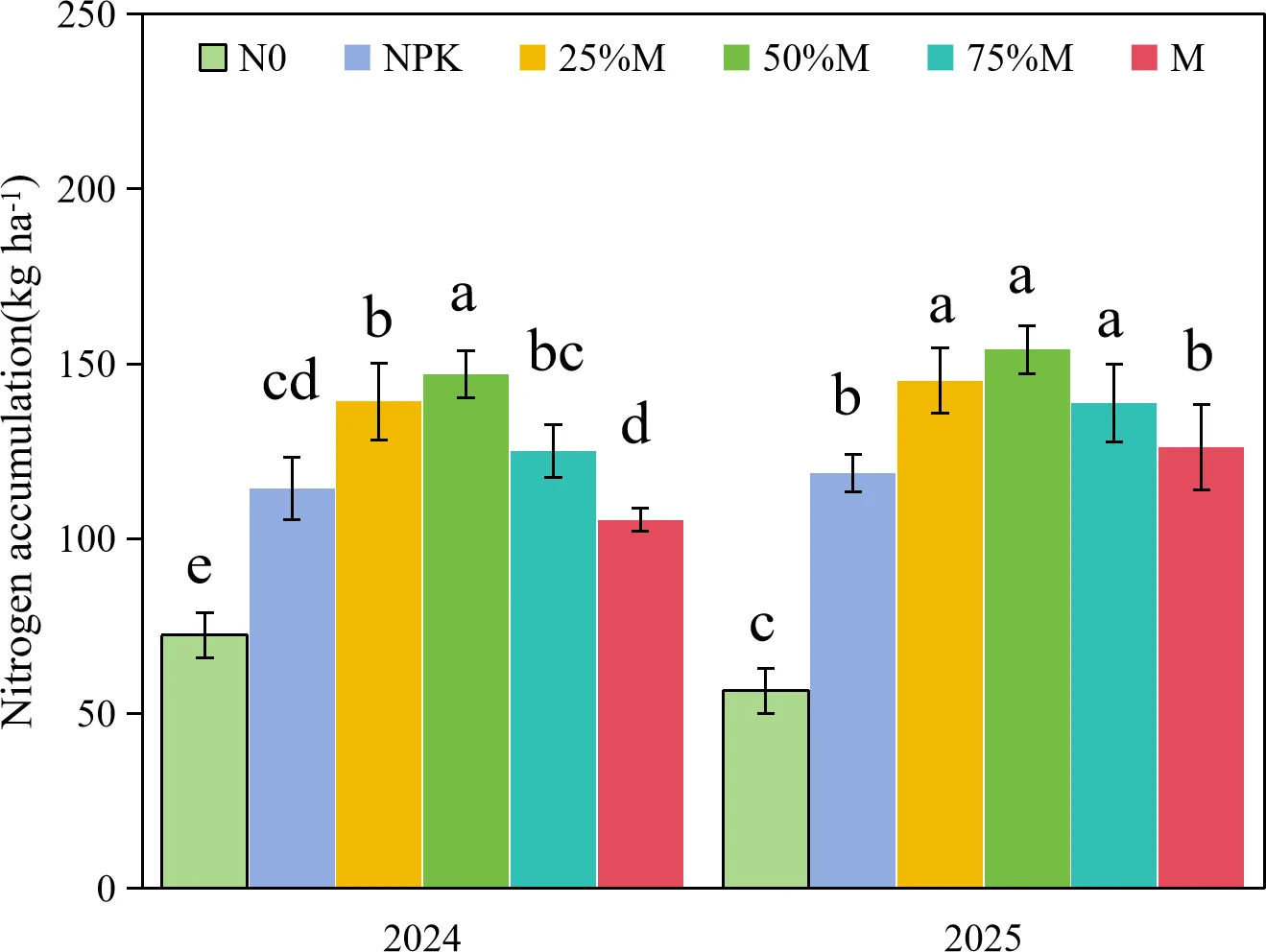
Effect of different organic substitution ratios on nitrogen accumulation in the above-ground parts of rice in soda saline-alkali soil. Values are the means with standard deviations shown by vertical bars (n = 3). N0 represents no nitrogen fertilizer treatment; M represents organic fertilizer replacing 25% N; 50% M represents organic fertilizer replacing 50% N; 75% M represents organic fertilizer replacing 75% N; M treatment represents complete application of organic fertilizer; The lowercase letters “a, b, c, d” indicate significant differences (*P*< 0.05) between different treatments.

### 2.4 Effect of different organic substitution ratios on nitrogen use in rice

The apparent nitrogen utilization efficiency, nitrogen agronomic efficiency, nitrogen partial productivity, and harvest index can reflect the utilization of nitrogen by crops (**Table 2**). Over the course of two years, different organic substitution treatments, except for treatment M, significantly improved the apparent nitrogen utilization efficiency, nitrogen agronomic efficiency, nitrogen partial productivity, and harvest index of rice compared to NPK treatment, with an average increase of 10.3-12.4, 2.2-2.9, 2.0-2.9, and 2.9-3.8 percentage points, respectively. In the organic substitution treatment, the rice with 50% M treatment had the highest apparent nitrogen utilization efficiency, nitrogen agronomic efficiency, nitrogen partial productivity, and harvest index, which increased by 7.8-10.8, 3.5-3.7, 3.4-3.5, and 3.4-4.9 percentage points, respectively, compared to other organic substitution treatments.

**Table 2:** Effect of different organic substitution ratios on nitrogen use in rice.

		Nitrogen utilization efficiency (%)	Nitrogen fertilizer productivity (kg/kg)	Agricultural efficiency (kg/kg)	Harvest index (%)
2024	N0	--	14.9 ± 1.0e	--	28.0 ± 1.1d
	NPK	19.1 ± 4.1cd	24.3 ± 0.8cd	9.5 ± 0.8c	33.0 ± 1.9bc
	25%M	27.3 ± 2.9ab	26.8 ± 2.0ab	12.0 ± 2.0ab	35.4 ± 3.4ab
	50%M	33.9 ± 3.1a	28.9 ± 1.4a	14.1 ± 1.4a	38.4 ± 0.6a
	75%M	23.9 ± 3.4bc	25.9 ± 1.2bc	11.1 ± 1.2bc	34.0 ± 2.4bc
	M	15.0 ± 1.5d	23.5 ± 0.7d	8.7 ± 0.7c	31.1 ± 2.1cd
2025	N0	--	13.4 ± 1.5c		27.9 ± 5.6c
	NPK	24.8 ± 3.9c	28.1 ± 0.7b	13.3 ± 0.7ab	32.8 ± 1.4bc
	25%M	30.6 ± 2.7a	29.8 ± 3.2ab	14.9 ± 2.9ab	34.6 ± 1.3ab
	50%M	34.0 ± 0.3a	32.1 ± 1.9a	16.9 ± 1.7a	38.6 ± 0.9a
	75%M	32.4 ± 1.6ab	28.9 ± 2.8ab	14.0 ± 2.5ab	36.9 ± 0.9ab
	M	24.1 ± 2.5c	26.4 ± 0.6b	11.8 ± 0.6b	34.1 ± 3.9ab

Values are the means with standard deviations shown by vertical bars (n = 3). N0 represents no nitrogen fertilizer treatment; NPK treatment represents inorganic nitrogen fertilizer treatment; 25% M represents organic fertilizer replacing 25% N; 50% M represents organic fertilizer replacing 50% N; 75% M represents organic fertilizer replacing 75% N; M treatment represents complete application of organic fertilizer; The lowercase letters “a, b, c, d” indicate significant differences (*P*< 0.05) between different treatments.

### 2.5 Effect of different organic substitution ratios on the accumulation of inorganic nitrogen in soil

#### 2.5.1 Effect of different organic substitution ratios on inorganic nitrogen accumulation in mature soil

As shown in **Figure 4**, The research results showed that the inorganic nitrogen accu mulation in the 0–100 cm soil layer before sowing in the experi mental field was 122.4 and 125.9 kg ha^-1^, respectively, over two years. However, the inorganic nitrogen accumulation in rice under various treatments after harvest decreased significantly compared to before sowing. There are certain differences in the total accumula tion of inorganic nitrogen in the 0^-1^00cm soil layer among different organic substitution treatments. The treatment without nitrogen application has the lowest accumulation, while the NPK treatment has the highest accumulation of inorganic nitrogen in the soil (65.3-84.3kg ha^-1^). The soil inorganic nitrogen accumulation was lowest when treated with 50% M (49.8-54.6kg ha^-1^). The vertical distribu tion of inorganic nitrogen in the soil treated for several days shows agradually decreasing trend from the surface layer to the deep layer. The results showed that organic substitution resulted in a decrease in soil inorganic nitrogen accumulation compared to NPK treat ment, which in turn affected the residual inorganic nitrogen in the soil after harvest, thereby reducing nitrogen residue and loss.

**Figure 4:**
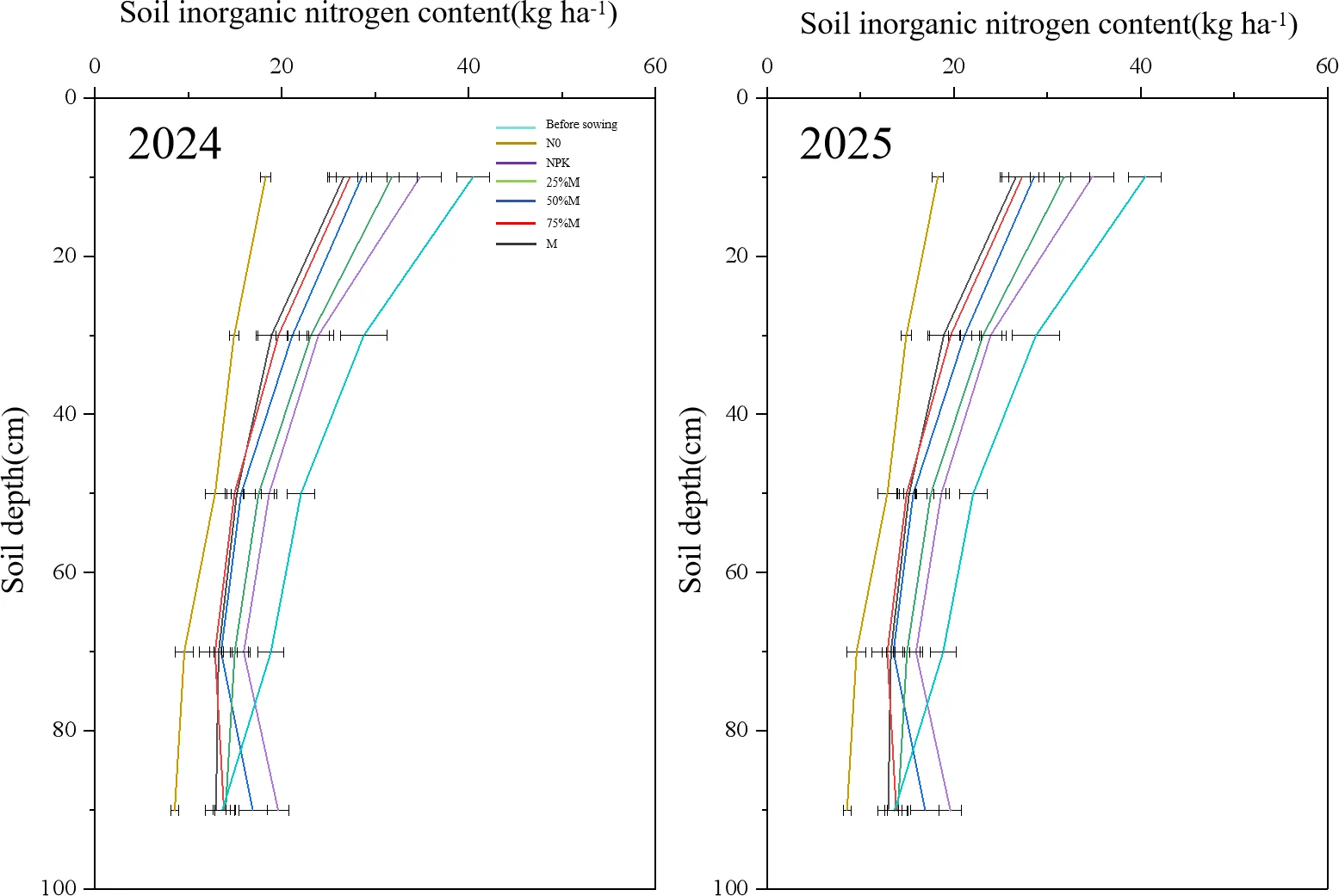
Effect of different organic substitution ratios on inorganic nitrogen accumulation in soil during maturity of rice in soda saline-alkali soil. Values are the means with standard deviations shown by vertical bars (n = 3). N0 represents no nitrogen fertilizer treatment; M represents organic fertilizer replacing 25% N; 50% M represents organic fertilizer replacing 50% N; 75% M represents organic fertilizer replacing 75% N; treatment represents the complete application of organic fertilizer.

#### 2.5.2 Effect of different organic substitution ratios on soil apparent nitrification rate

From **Figure 5**, it can be seen that there are significant differences in the apparent nitrification rate of soil among different treatments over two years. Organic substitution of inorganic fertilizers can significantly reduce the apparent nitrification rate of soil and inhibit the growth rate of soil apparent nitrification rate in the later stage. Compared with NPK treatment, each organic substitution treatment decreased by 11.3% -15.7%, 22.1% -31.5%, 20.5% -38.0%, and 30.3% -35.4% at different stages. In organic substitution treatment, the 50% M treatment had the lowest apparent nitrification rate during the mature stage, with an average reduction of 9.8% -15.5% compared to other organic substitution treatments. Organic substitution can effectively inhibit soil nitrification and delay the conversion of ammonium nitrogen to nitrate nitrogen.

**Figure 5:**
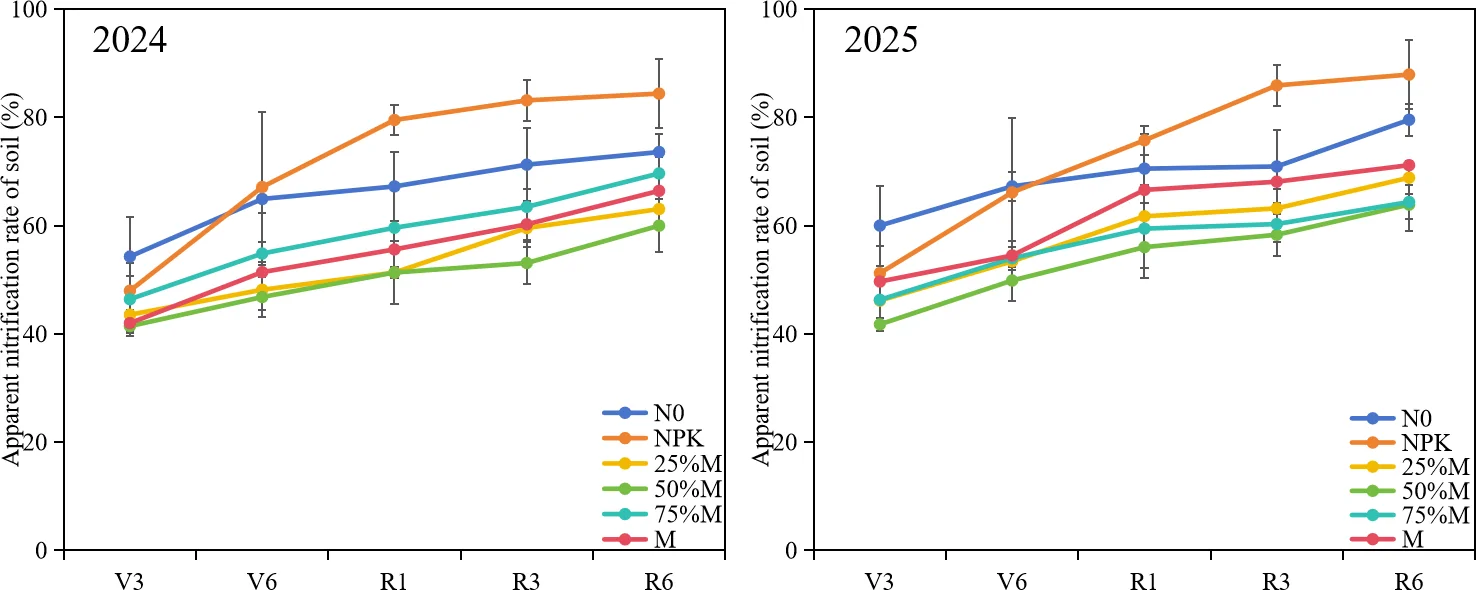
Effect of different organic substitution ratios on apparent nitrification rate of soil during rice growth in soda saline-alkali soil. V3 represents the three leaf stage; V6 represents the six leaf stage; R1 represents the tassel germination stage (heading stage); R3 represents the milk ripening period; R6 represents the fully matured stage. Leaf period.Values are the means with standard deviations shown by vertical bars (n = 3). N0 represents no nitrogen fertilizer treatment; NPK treatment represents inorganic nitrogen fertilizer treatment; 25% M represents organic fertilizer replacing 25% N; 50% M represents organic fertilizer replacing 50% N; 75% M represents organic fertilizer replacing 75% N; M treatment represents complete application of organic fertilizer.

### 2.6 Effect of different organic substitution ratios on nitrogen loss in rice

#### 2.6.1 Effect of different organic substitution ratios on ammonia volatilization flux

The volatilization rate of NH_3_ from rice in soda saline alkali soil under different organic substitution treatments is shown in **Figure 6**. During the two years, the NH_3_ volatilization rate first increased rapidly, then reached a peak, and then began to decrease. In the high temperature weather after rainstorm, the NH_3_ volatilization rate would have a fast peak, then decreased rapidly, repeated several times, and finally stabilized. The volatilization rate of NH_3_ in N0 treatment was the lowest, consistently lower than that of each nitrogen application treatment. The second peak of NH_3_ volatilization rate appeared on the 6th day after fertilization, and the peak values of NPK treatment were 4.3-6.2 kg ha^-1^ d^-1^, which were 1.6-2.1 kg ha^-1^ d^-1^ higher than those of N0 treatment. After the peak, the volatilization rate of NH_3_ in the nitrogen application treatment gradually decreased, and on the 41st day after fertilization, it dropped to slightly higher levels than the non fertilization treatment. On the 5th day after fertilization, the highest NH_3_ volatilization rate was observed in all nitrogen treatments, with the highest value reaching 2.5-3.9kg ha^-1^ d^-1^ in the 50% M treatment, which was 1.0-1.8kg ha^-1^ d^-1^ higher than the N0 treatment. Subsequently, the volatilization rate of NH_3_ in each treatment gradually stabilized until there was no significant difference.

**Figure 6:**
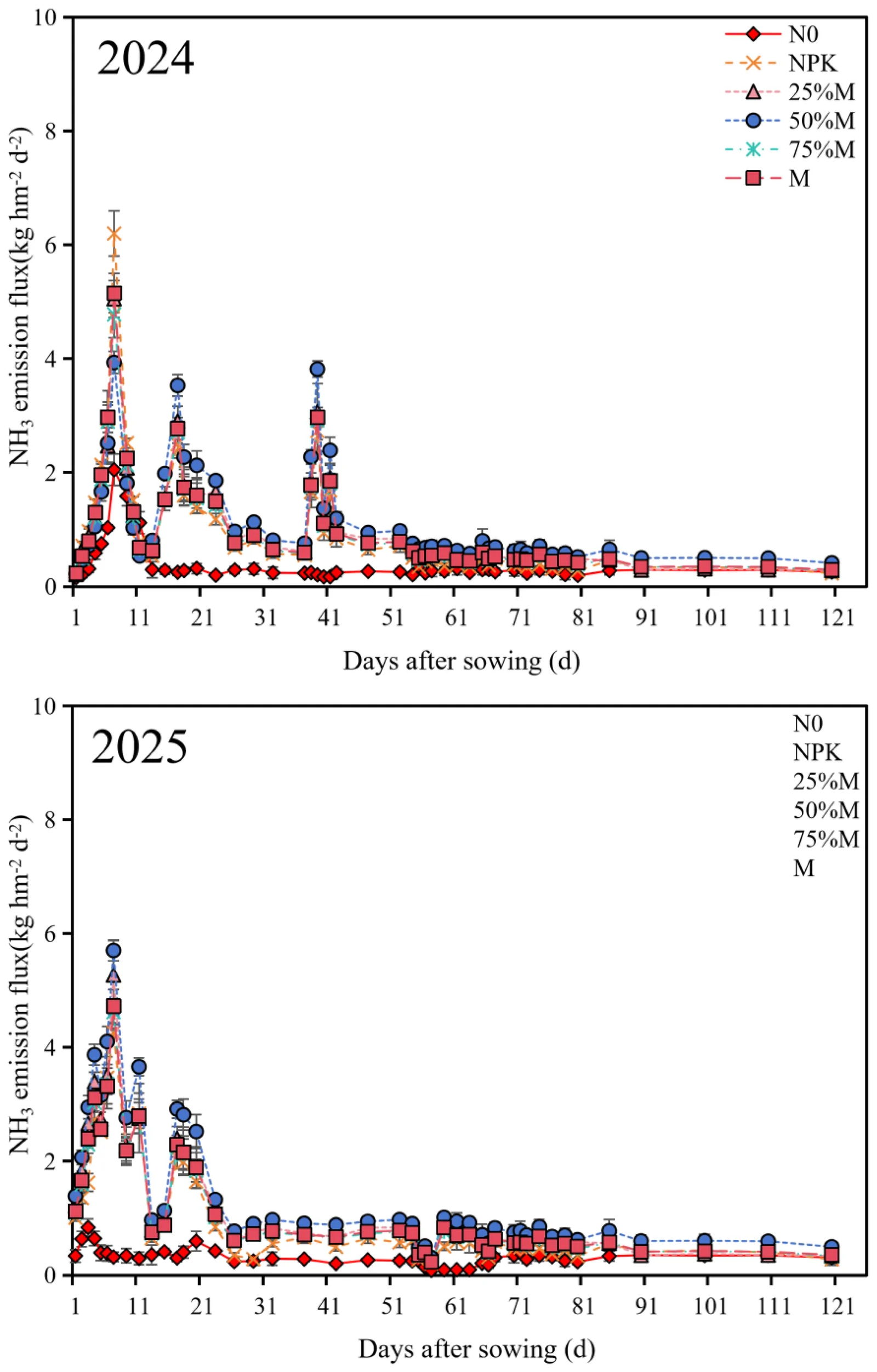
Effect of different organic substitution ratios on ammonia volatilization flux of rice in soda saline alkali soil. Values are the means with standard deviations shown by vertical bars (n = 3). N0 represents no nitrogen fertilizer treatment; NPK treatment represents inorganic nitrogen fertilizer treatment; 25% M represents organic fertilizer replacing 25% N; 50% M represents organic fertilizer replacing 50% N; 75% M represents organic fertilizer replacing 75% N; treatment represents the complete application of organic fertilizer.

#### 2.6.2 Effect of different organic substitution ratios on Cumulative ammonia volatilization emissions

The cumulative volatilization of NH_3_ from rice in soda saline alkali soil under different organic substitution treatments is shown in **Figure 7**. Without considering nitrogen treatment, the average NH_3_ volatilization accumulation in 2025 is 38.73 kg ha^-1^, an increase of 11.15 kg ha^-1^ compared to 2024. Regardless of the experimental year, the cumulative amount of soil NH_3_ volatilization in each treatment is always higher than that in the N0 treatment, showing an upward trend. Compared with NPK treatment, each organic substitution treatment reduced the average NH_3_ volatilization accumulation by 12.3-19.98%. Among the various organic substitution treatments, the NH_3_ volatilization accumulation in the 50% M treatment was the lowest, with an average reduction of 8.9% -11.1% compared to other organic substitution treatments.

**Figure 7:**
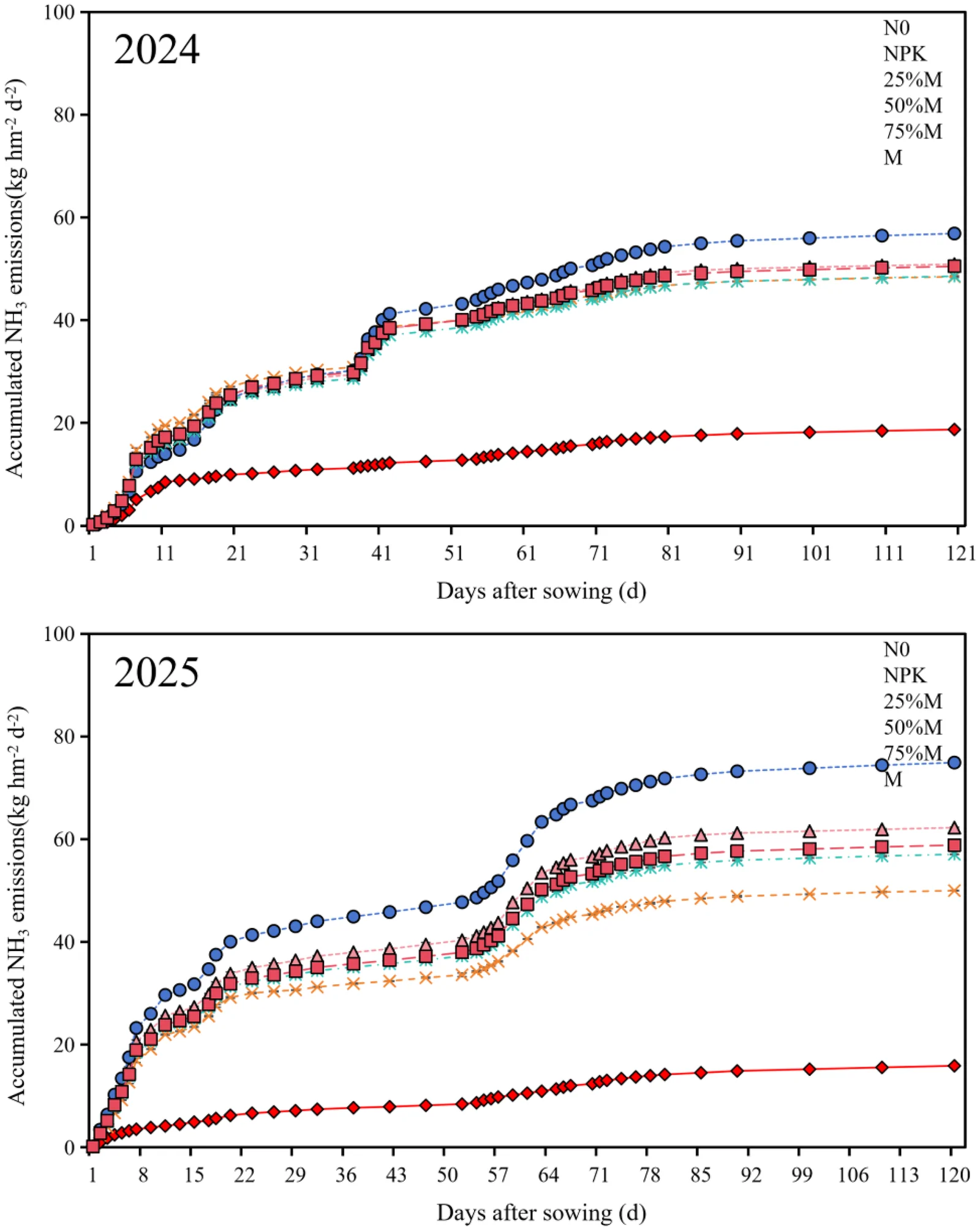
Effect of different organic substitution ratios on the ammonia volatilization, emission and accumulation of rice in soda saline alkali soil. Note: Values are the means with standard deviations shown by vertical bars (n = 3). N0 represents no nitrogen fertilizer treatment; NPK treatment represents inorganic nitrogen fertilizer treatment; 25% M represents organic fertilizer replacing 25% N; 50% M represents organic fertilizer replacing 50% N; 75% M represents organic fertilizer replacing 75% N; treatment represents the complete application of organic fertilizer.

#### 2.6.3 Effect of different organic substitution ratios on N_2_O emission rate

The effects of different organic substitution treatments on the volatilization rate of N_2_O from rice in soda saline alkali soil are shown in **Figure 8**. In this study, the emission flux of N_2_O from soil is closely related to soil fertility and precipitation. The trend of N_2_O emission flux on saline alkali soil in 2024 and 2025 is basically consistent. Compared to the non-nitrogen treatment, the nitrogen treatment significantly increased N_2_O emission flux. Over the course of two years, N0 treatment maintained relatively low N_2_O emission fluxes. The average N_2_O emission flux was highest in the NPK treatment among all nitrogen application treatments, because the NPK treatment had a higher urea input and a larger N_2_O emission flux. The average N_2_O emission flux of NPK treatment is slightly higher than that of organic substitution treatment, because compared with organic substitution treatment, urea in NPK treatment caused a larger N_2_O emission flux in the early stage, resulting in an increase in the average N_2_O emission flux. On the 7th day after fertilization in 2024, the NPK, 25% M, 50% M, 75% M, and M treatments all showed sub peak N_2_O emission fluxes of 46.5, 35.7, 30.3, 25.5, and 21.2 µg m^-2^ h^-1^, respectively. The peak of N_2_O emission flux for each fertilization treatment in 2025 also occurred on the 7th day after fertilization, at 51.2, 45.5, 40.9, 32.9, and 22.1 µg m^-2^ h^-1^, respectively. During the two-year period, there will be three small peaks in N_2_O emissions accompanied by continuous high temperatures after rainfall, all of which are significantly smaller than the first two peaks and gradually stabilize until there is no significant difference.

**Figure 8:**
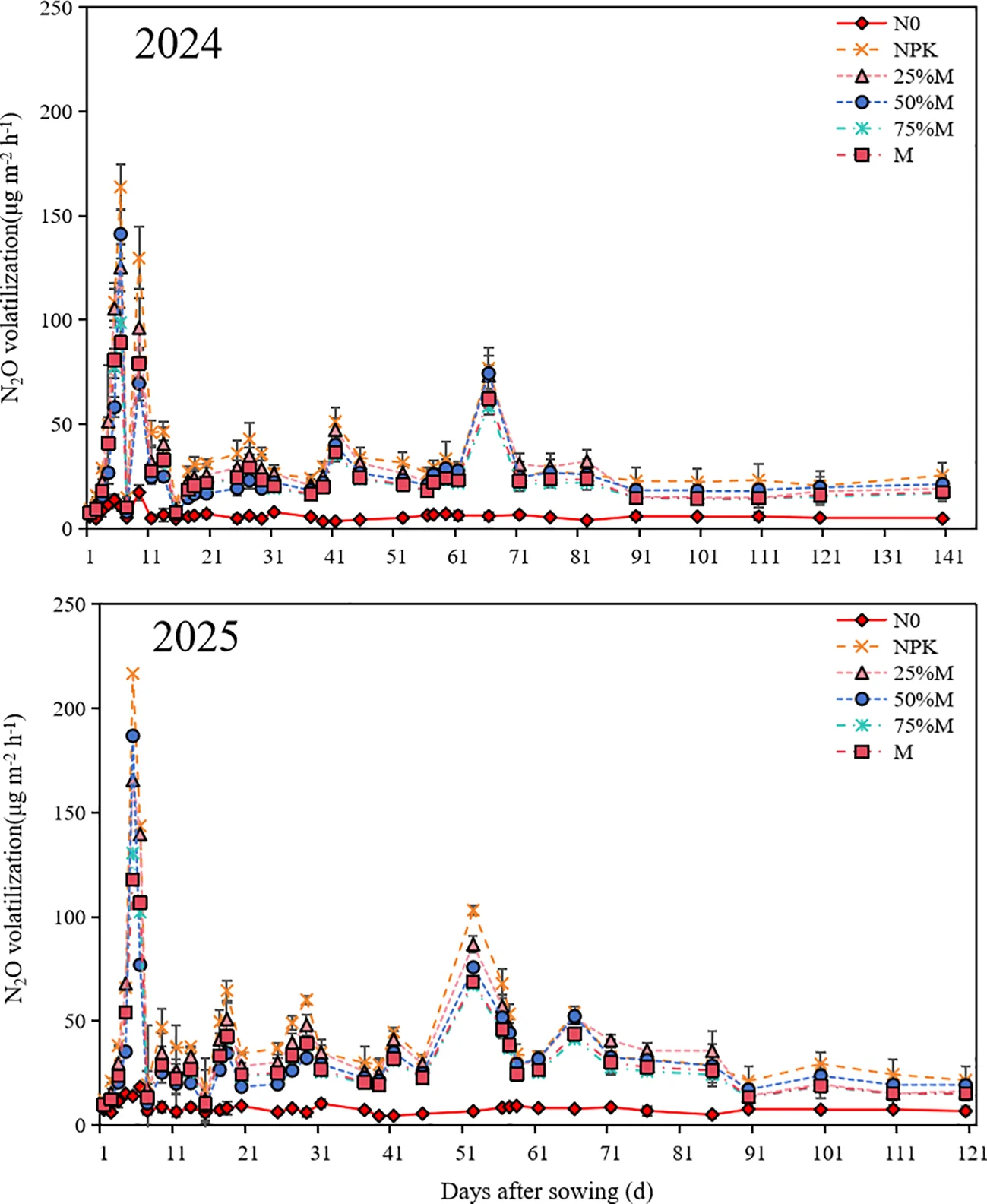
The effect of different organic substitution ratios on N_2_O emission rate from rice in soda saline alkali soil. Values are the means with standard deviations shown by vertical bars (n = 3). N0 represents no nitrogen fertilizer treatment; M represents organic fertilizer replacing 25% N; 50% M represents organic fertilizer replacing 50% N; 75% M represents organic fertilizer replacing 75% N; M treatment represents complete application of organic fertilizer.

#### 2.6.4 Effect of different organic substitution ratios on N_2_O emissions accumulated

The effects of different organic substitution treatments on the volatilization and accumulation of N_2_O in rice grown in soda saline alkali soil are shown in **Figure 9**. In this study, as the number of days increased, the cumulative volatilization of N_2_O in each treatment showed a gradual and slow increase. The trend of N_2_O emission flux on black soil and sandy soil in 2024 and 2025 is basically consistent. Over the course of two years, N0 treatment maintained relatively low N_2_O emission fluxes. Compared to the non-nitrogen treatment, the nitrogen treatment significantly increased the cumulative N_2_O emissions, with an average increase of 35.1% -45.5%. Among all nitrogen treatments, the average N_2_O emission flux was highest in the NPK treatment. This is because the NPK treatment had a high urea input, resulting in a larger N_2_O emission flux and the highest cumulative N_2_O emissions throughout the entire growth period. The average N_2_O emission flux of NPK treatment was significantly higher than that of organic substitution treatment, with an average increase of 23.1% -25.5% over two years. Among the various organic substitution treatments, the M treatment had the smallest cumulative N_2_O emissions, but there was no difference compared to the 50% M and 75% M treatments.

**Figure 9:**
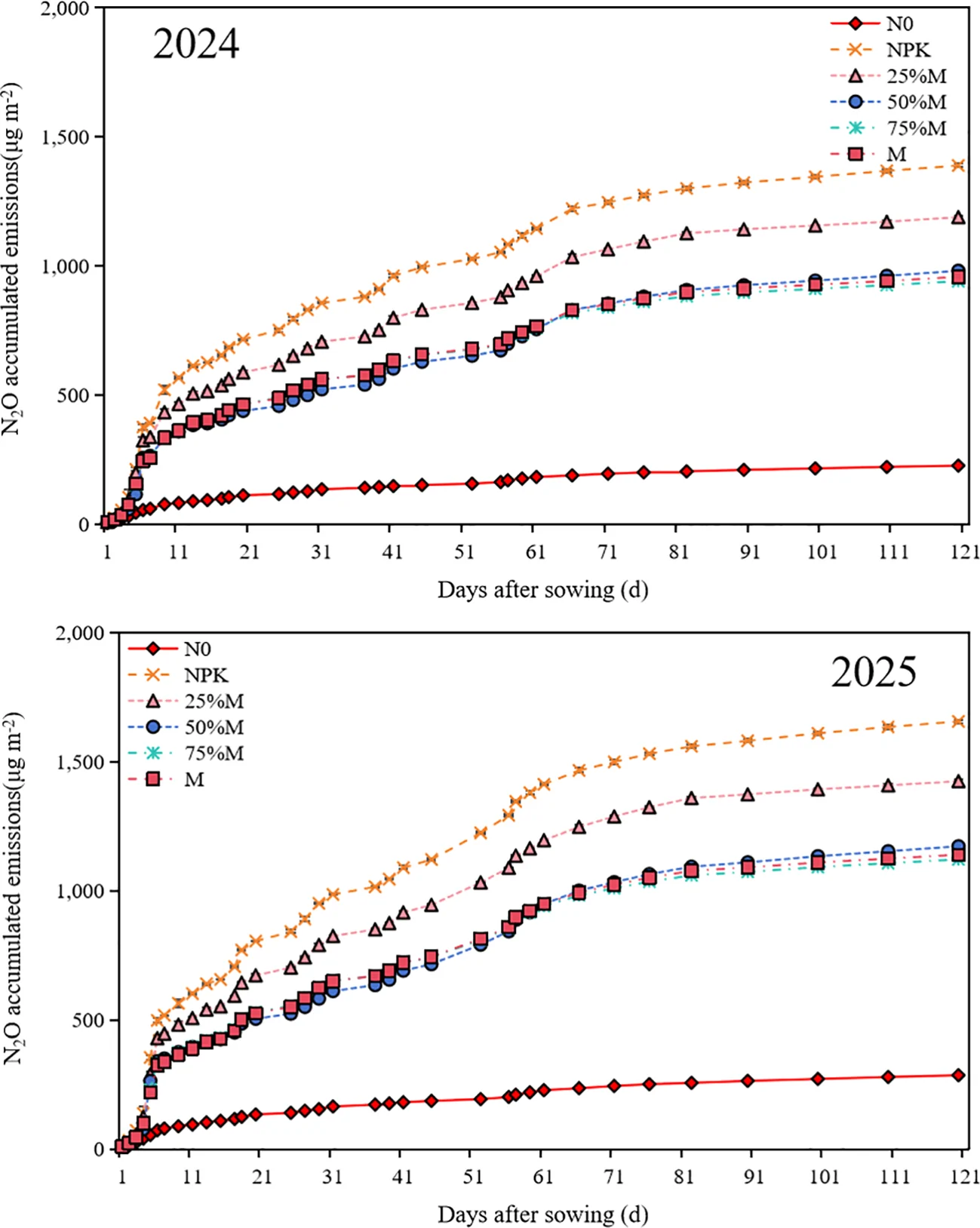
Effect of different organic substitution ratios on the cumulative N2O emissions from rice in soda saline alkali soil. Values are the means with standard deviations shown by vertical bars (n = 3). N0 represents no nitrogen fertilizer treatment; N; 50% M represents organic fertilizer replacing 50% N; 75% M represents organic fertilizer replacing 75% N; M treatment represents complete application of organic fertilizer.

#### 2.6.5 effect of different organic substitution ratios on CH_4_ emissions

The effects of different organic substitution treatments on CH_4_ emission flux from rice in soda saline alkali soil are shown in **Figure 10**. The trend of CH_4_ emission flux on black soil and sandy soil in 2024 and 2025 is basically consistent. Compared to the non-nitrogen treatment, the nitrogen treatment significantly increased CH_4_ emission flux. Over the course of two years, N0 treatment maintained relatively low CH_4_ emission fluxes. The average CH_4_ emission flux of NPK treatment is significantly lower than that of organic substitution treatment. In 2024 and 2025, on the 7th day after fertilization, NPK, 25% M, 50% M, 75% M, and M treatments all showed peak CH_4_ emission fluxes of 46.5, 35.7, 30.3, 25.5, and 21.2 µg m^-2^ h^-1^, respectively. The peak of CH_4_ emission flux for each fertilization treatment in 2025 also occurs on the 7th day after fertilization. The peak to peak CH_4_ emission flux of organic substitution treatment increased by an average of 23.1% -28.9% compared to NPK treatment. The peak to peak CH_4_ emission flux of M treatment is the highest in organic substitution treatment, with an average increase of 11.1% -15.3% compared to other organic substitution treatments.

**Figure 10:**
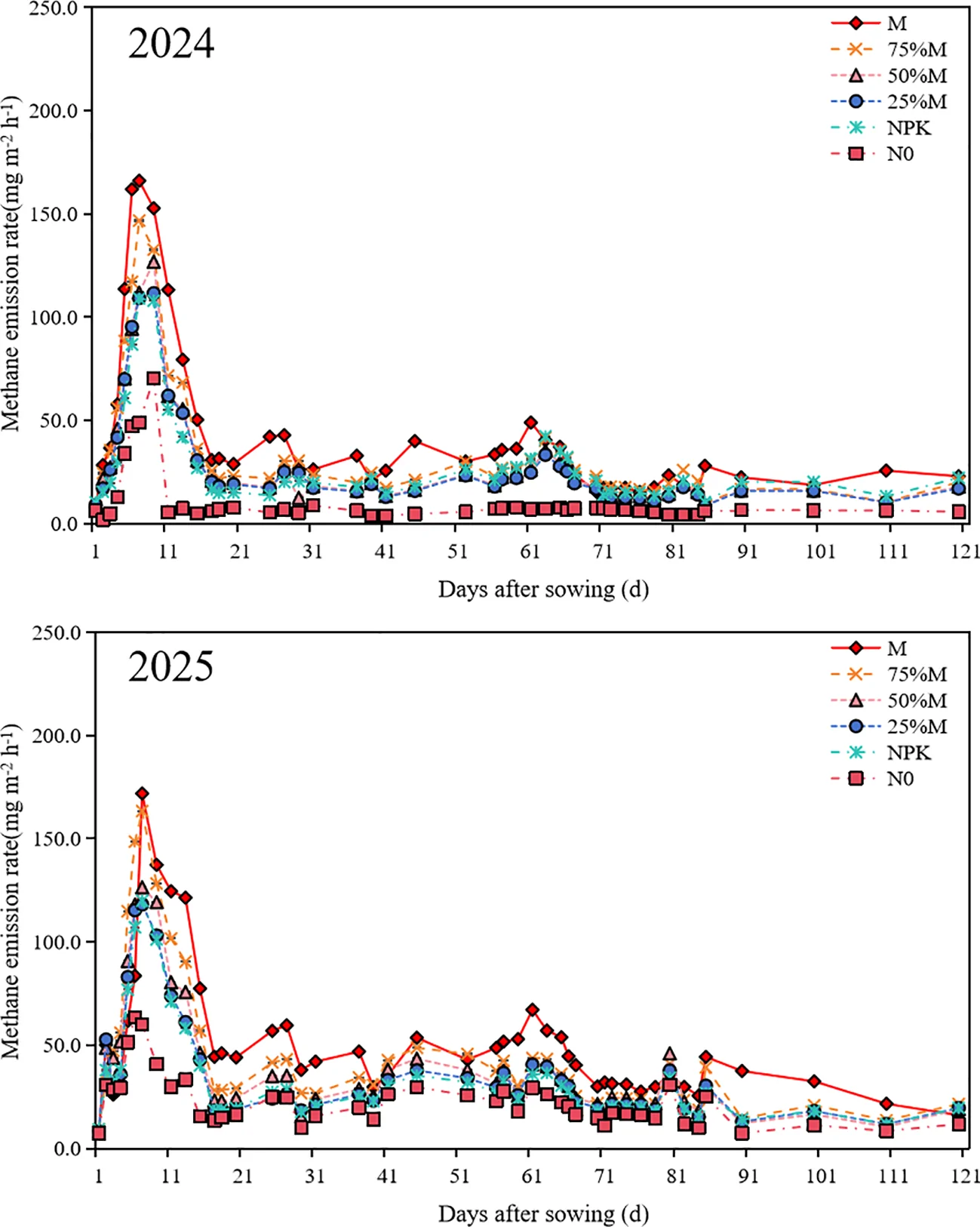
The effect of different organic substitution ratios on CH_4_ emission rate from rice in soda saline alkali soil. Values are the means with standard deviations shown by vertical bars (n = 3). N0 represents no nitrogen fertilizer treatment; M represents organic fertilizer replacing 25% N; 50% M represents organic fertilizer replacing 50% N; 75% M represents organic fertilizer replacing 75% N; M treatment represents complete application of organic fertilizer.

### 2.7 Effect of different organic substitution ratios on nitrogen fate in rice

As shown in **Table 3**, Over the course of two years, the NPK treatment resulted in the lowest apparent utilization rate of nitrogen fertilizer, the highest apparent residual rate, and the highest apparent loss rate of nitrogen fertilizer. The apparent utilization efficiency of nitrogen fertilizer in organic substitution treatment was significantly higher than that in NPK treatment. In organic substitution treatment, the apparent utilization rate of nitrogen fertilizer is highest in the 50% M treatment, and the apparent residual rate of nitrogen fertilizer is lowest in NPK. Therefore, the apparent loss rate of nitrogen fertilizer is lowest in the 50% M treatment. The results indicate that organic substitution treatment can significantly reduce the apparent loss of nitrogen fertilizer and improve nitrogen fertilizer utilization efficiency, resulting in a higher nitrogen fertilizer utili zation efficiency.

**Table 3:** Effect of different organic substitution ratios on nitrogen fate in rice.

		Nitrogen fertilizer utilization rate (%)	Nitrogen residue rate (%)	Nitrogen loss rate (%)
2024	N0	--	--	--
	NPK	19.1 ± 4.1ab	17.6 ± 1.2c	63.4 ± 4.8a
	25%M	30.4 ± 5.0a	19.9 ± 2.0bc	49.8 ± 6.6a
	50%M	33.9 ± 3.1a	21.4 ± 2.4ab	44.7 ± 2.6b
	75%M	23.9 ± 3.4ab	21.9 ± 2.1ab	54.3 ± 1.3bc
	M	15.0 ± 1.5b	24.3 ± 0.9a	60.7 ± 2.0c
2025	N0	--	--	--
	NPK	28.3 ± 2.5c	17.5 ± 3.0b	54.2 ± 2.9a
	25%M	40.3 ± 4.3a	21.4 ± 1.1ab	38.3 ± 4.0b
	50%M	44.3 ± 3.2a	23.3 ± 1.7a	32.4 ± 4.8b
	75%M	37.4 ± 5.0ab	23.1 ± 1.9a	39.5 ± 5.2b
	M	31.7 ± 5.5bc	24.5 ± 2.6a	43.8 ± 6.7b

Values are the means with standard deviations shown by vertical bars (n = 3). N0 represents no nitrogen fertilizer treatment; NPK treatment represents inorganic nitrogen fertilizer treatment; 25% M represents organic fertilizer replacing 25% N; 50% M represents organic fertilizer replacing 50% N; 75% M represents organic fertilizer replacing 75% N; M treatment represents complete application of organic fertilizer; The lowercase letters “a, b, c, d” indicate significant differences (*P*< 0.05) between different treatments.

## 3 Discussion

Soda saline-alkali land severely restricts rice growth and yield due to its high pH, high salt content, and low organic matter content (Liu et al., 2022; Wei et al., 2022; Wang et al., 2024). As an effective measure to ameliorate saline-alkali land and optimize crop production, the combined application of organic and inorganic fertilizers achieves synergistic adaptation between soil environment improvement and crop growth requirements (Rezapour et al., 2023; Li et al., 2020). A key finding of this study is that the organic-inorganic combined treatment significantly increased rice yield and aboveground dry matter accumulation, with the 50% organic nitrogen substitution ratio (50%M) consistently exhibiting the best performance in both 2024 and 2025. This result is consistent with previous studies on the application of organic fertilizers in saline-alkali land to improve crop productivity.The consistent superiority of the 50%M treatment in rice yield is mainly attributed to its optimal regulation of soil conditions, nutrient supply, and root growth. Firstly, the abundant organic matter in organic fertilizers is decomposed by microorganisms into humus, which effectively improves the soil structure of soda saline-alkali land, reduces bulk density, enhances permeability, and creates a favorable physical environment for rice root growth (Liu et al., 2020; Shankar et al., 2022). Secondly, humus buffers soil pH through its adsorption and ion exchange properties, alleviates sodium ion toxicity to roots, and promotes root water and nutrient uptake (Olaetxea et al., 2021). Thirdly, organic nitrogen components such as amino acids and peptides released by organic fertilizers form a synergistic supply with fertilizer nitrogen, avoiding nutrient imbalance caused by a single nitrogen source and matching the nitrogen demand pattern of rice at different growth stages, thereby promoting the synthesis of photosynthetic products and laying a material foundation for dry matter accumulation and yield formation (Deng et al., 2019; Cheng et al., 2025). In contrast, the total organic nitrogen treatment (M treatment) failed to show yield advantages, as the slow mineralization of organic nitrogen could not meet the critical nitrogen demand of rice during key growth periods, resulting in a mismatch between nutrient supply and crop growth rhythm. This confirms that the organic nitrogen substitution ratio must be controlled within a reasonable range, with 50% being the optimal proportion.

Nitrogen use efficiency, a key indicator of fertilization rationality (Cheng et al., 2025), was significantly improved by the combined application of organic and inorganic fertilizers, with the 50%M treatment achieving the highest efficiency and the single chemical fertilizer treatment (NPK) the lowest. This is consistent with previous studies on saline-alkali and other soil types, confirming the universal applicability of this measure. The core mechanism lies in optimizing soil nitrogen cycling: organic matter adsorbs inorganic nitrogen to reduce losses through leaching and volatilization (Xing et al., 2025), promotes the proliferation of nitrifying and nitrogen-fixing microorganisms to enhance nitrogen transformation (Tang et al., 2021), and improves rice nutritional status to optimize nitrogen transport and distribution (Ly et al., 2015). Different substitution ratios had distinct effects: low (25%M) substitution had a limited improvement on soil structure and nitrogen supply, while high (75%M) and total (M) substitution could not meet the rapid nitrogen demand of rice and even affected root respiration due to organic matter decomposition, thus reducing nitrogen use efficiency. This further verifies that the 50% substitution ratio achieves the optimal balance between organic and inorganic nitrogen, providing a key basis for rational fertilization in soda saline-alkali land.Soil nitrogen residue, an important indicator of fertilization environmental risk (Ren et al., 2011; Li et al., 2020; Zhao et al., 2024), was significantly reduced by the combined application of organic and inorganic fertilizers, with the 50%M treatment having the lowest residue and the NPK treatment the highest, which is consistent with previous research conclusions. This is because the 50%M treatment improves rice nitrogen absorption and utilizes organic matter to fix inorganic nitrogen, reducing nitrogen mobility and deep leakage (Dittert et al., 2005; Id et al., 2020; Gautam et al., 2021). Additionally, it reduces the soil apparent nitrification rate, delaying the conversion of ammonium nitrogen to nitrate nitrogen (the main form of nitrogen leaching) (Ma et al., 1999; Compton et al., 2000; Pasda et al., 2001), thereby reducing the leaching risk. Nitrogen residue was highest in the 0–20 cm surface layer and decreased with soil depth, which is related to the fertilization method and soil physicochemical properties. Notably, compared with the NPK treatment, the 50%M treatment significantly reduced nitrogen residue in deep soil (60–100 cm), effectively reducing the risk of groundwater pollution and supporting the green agricultural development of soda saline-alkali land.

Nitrogen loss, a major cause of low fertilizer efficiency and environmental pollution, was significantly mitigated by the combined application of organic and inorganic fertilizers, with the 50%M treatment having the lowest loss and the NPK treatment the highest, which is consistent with previous studies in saline-alkali and paddy ecosystems (Wang et al., 2024; Bhattarai et al., 2025). For ammonia volatilization (a key nitrogen loss pathway in soda saline-alkali paddy fields), the combined application of organic and inorganic fertilizers reduces the ammonium nitrogen concentration in the soil solution through humus adsorption (Kohira et al., 2024), regulates soil pH through the production of organic acids (Clemens et al., 2002), and improves soil water and fertilizer retention to reduce surface fertilizer accumulation (Schlüter, 2011), thus suppressing volatilization. For N_2_O (an important greenhouse gas), the 50%M treatment optimizes nitrogen supply to avoid excessive soil nitrogen accumulation, promotes microbial nitrogen assimilation, and reduces N_2_O emissions derived from nitrification and denitrification (Ameloot et al., 2013; Venterea and Coulter, 2015).A notable phenomenon is that the combined application of organic and inorganic fertilizers increased CH_4_ emission flux while reducing N_2_O emissions, with this effect enhancing with the increase in organic nitrogen substitution ratio. The peak CH_4_ emissions of the organic substitution treatments were 23.1%-28.9% higher than those of the NPK treatment, and the M treatment was 11.1%-15.3% higher than the 50%M treatment. This is because organic fertilizers (e.g., pig manure) provide abundant organic carbon, which is decomposed to supply energy for methanogens, thereby promoting their activity and proliferation (Krüger and Frenzel, 2010); the improved soil aeration combined with flooded anaerobic conditions further exacerbates CH_4_ emissions. Although the 50%M treatment increased CH_4_ emissions compared with the NPK treatment, its comprehensive greenhouse effect is still lower due to the significant reductions in ammonia volatilization, N_2_O emissions, and soil nitrogen residue. Future research should focus on balancing the impacts of organic-inorganic application on different greenhouse gases, such as optimizing the substitution ratio and water management to reduce CH_4_ emissions, and achieving a win-win situation between agricultural production and environmental protection.

## 4 Conclusion

Under the condition of organic-inorganic combined application in soda saline-alkali soil, the appropriate proportion of organic nitrogen replacing inorganic nitrogen (50% M treatment) improved rice yield and nitrogen use efficiency while reducing nitrogen loss and environmental risks. Its mechanism involves optimizing soil physical and chemical properties and structure through organic matter decomposition and humus formation, enhancing soil water and fertilizer retention capacity and root nutrient absorption capacity, promoting rice aboveground dry matter accumulation and nitrogen accumulation; regulating soil nitrogen transformation process, reducing apparent nitrification rate, slowing down ammonium nitrogen conversion to nitrate nitrogen, decreasing inorganic nitrogen residue in soil and deep migration, and reducing nitrogen leaching risk; mitigating nitrogen loss pathways such as ammonia volatilization and N_2_O emission by adsorbing ammonium nitrogen and regulating soil pH, improving nitrogen apparent utilization rate and reducing nitrogen loss rate, thus realizing the coordination of rice yield increase, nitrogen efficient utilization and environmental protection. In addition, excessive or insufficient organic nitrogen substitution ratio cannot achieve the optimal effect, and the balanced supply of organic and inorganic nitrogen is the key to adapting to the growth demand of rice in soda saline-alkali soil and achieving green and sustainable production.

**Table:** .

## Data Availability

The original contributions presented in the study are included in the article/supplementary material. Further inquiries can be directed to the corresponding author.
